# The Specificity of Cognitive-Motor Dual-Task Interference on Balance in Young and Older Adults

**DOI:** 10.3389/fnagi.2021.804936

**Published:** 2022-01-11

**Authors:** Nathan Ward, Alekya Menta, Virginia Ulichney, Cristiana Raileanu, Thomas Wooten, Erika K. Hussey, Elizabeth Marfeo

**Affiliations:** ^1^Department of Psychology, Tufts University, Medford, MA, United States; ^2^Department of Psychology, Temple University, Philadelphia, PA, United States; ^3^Defense Innovation Unit, Mountain View, CA, United States; ^4^Department of Occupational Therapy, Tufts University, Medford, MA, United States

**Keywords:** cognitive-motor multitasking, dual-tasking, executive function, aging, multitasking ability

## Abstract

Standing upright on stable and unstable surfaces requires postural control. Postural control declines as humans age, presenting greater risk of fall-related injury and other negative health outcomes. Secondary cognitive tasks can further impact balance, which highlights the importance of coordination between cognitive and motor processes. Past research indicates that this coordination relies on executive function (EF; the ability to control, maintain, and flexibly direct attention to achieve goals), which coincidentally declines as humans age. This suggests that secondary cognitive tasks requiring EF may exert a greater influence on balance compared to non-EF secondary tasks, and this interaction could be exaggerated among older adults. In the current study, we had younger and older adults complete two Surface Stability conditions (standing upright on stable vs. unstable surfaces) under varying Cognitive Load; participants completed EF (Shifting, Inhibiting, Updating) and non-EF (Processing Speed) secondary cognitive tasks on tablets, as well as a single task control scenario with no secondary cognitive task. Our primary balance measure of interest was sway area, which was measured with an array of wearable inertial measurement unit sensors. Replicating prior work, we found a main effect of Surface Stability with less sway on stable surfaces compared to unstable surfaces, and we found an interaction between Age and Surface Stability with older adults exhibiting significantly greater sway selectively on unstable surfaces compared to younger adults. New findings revealed a main effect of Cognitive Load on sway, with the single task condition having significantly less sway than two of the EF conditions (Updating and Shifting) and the non-EF condition (Processing Speed). We also found an interaction of Cognitive Load and Surface Stability on postural control, where Surface Stability impacted sway the most for the single task and two of the executive function conditions (Inhibition and Shifting). Interestingly, Age did not interact with Cognitive Load, suggesting that both age groups were equally impacted by secondary cognitive tasks, regardless the presence or type of secondary cognitive task. Taken together, these patterns suggest that cognitive demands vary in their impact on posture control across stable vs. unstable surfaces, and that EF involvement may not be the driving mechanism explaining cognitive-motor dual-task interference on balance.

## Introduction

Research over the past several decades has found that standing upright involves multiple levels of controlled and automatic processing to integrate multiple streams of information (Peterka, 2002; Boisgontier et al., 2013). Biologically speaking, postural control involves interactions between cerebellar and cortical regions (Jacobs and Horak, 2007), as well as interactions among fronto-striatal regions (Mihara et al., 2008).

To complicate matters even further, humans often face situations in which they must maintain balance on unstable, irregular surfaces (e.g., an uneven sidewalk or a muddy patch of grass), which may require additional neural resources to avoid falls and injuries (Peterka, 2002; Agrawal et al., 2009). In line with this, research has found reduced postural control with decreased surface stability (Dault et al., 2001b; Bayot et al., 2018), with the impact of these physical demands varying as a function of specific surface stability manipulations (Barbado Murillo et al., 2012; Remaud et al., 2012; Lanzarin et al., 2015).

Postural control is not just impacted by physical demands but also by concurrent cognitive demands (Pellecchia, 2003; Costa et al., 2020), which further emphasizes the importance of cortical areas for standing upright (Woollacott and Shumway-Cook, 2002). One finds evidence for this in studies that require participants to maintain balance while performing cognitive tasks, which leads to impaired postural control (Lajoie et al., 1993; Andersson et al., 2002; Huxhold et al., 2006). This cognitive-motor interaction may be due to limitations in how humans use higher-order cognitive processing to manage the coordination of multiple tasks. For instance, task performance costs may come from bottlenecks in our information-processing architecture (Pashler, 1994; Borst et al., 2010) or from competition for limited attentional resources (Wickens, 2002). If task performance costs come from information-processing bottlenecks, then we expect to see general interference regardless the specific tasks; however, if the costs come from limited attentional resources, then we expect to see greater interference for tasks that require similar attentional resources.

Postural control is especially important for older adults who are at a higher risk of injury from falls (Fuller, 2000). In general, aging has been associated with postural and balance problems (Hageman et al., 1995; Laughton et al., 2003; Laufer et al., 2006; Ambrose et al., 2013), including declines in postural stability (Gill et al., 2001; Choy et al., 2003). Even without additional cognitive demands, healthy older adults tend to exhibit more postural sway than their younger counterparts (Kim et al., 2010). Furthermore, in cognitive-motor dual-task settings, older adults have demonstrated poorer balance and cognitive performance compared to younger adults (Schaefer, 2014), which has implications for daily activities and risk of falls (Lajoie and Gallagher, 2004; Beauchet et al., 2009).

Interestingly, age-related differences in cognitive-motor dual-task interference differ depending on the nature of the secondary cognitive task, especially when both the postural task and cognitive task recruit common neural resources that may atrophy as humans age (Rypma et al., 2001; Johnson et al., 2004; Fraizer and Mitra, 2008). For example, one study had older and younger adults verbally list words or type words while standing on stable or unstable surfaces and found differences in how the verbal and texting tasks impacted postural control across their age groups (Hsiao et al., 2020).

The current study builds on this line of research by further exploring the specificity of cognitive-motor dual-task interference in younger and older adults within the same modality. In the cognitive domain, we focused on executive function, which consists of higher cognitive processes important for controlling goal-directed behaviors (Garavan et al., 2000; Jurado and Rosselli, 2007; Chan et al., 2008; Bayot et al., 2018). Contemporary models suggest that executive function is made up of distinct but related components that allow humans to control, maintain, and flexibly direct attention to achieve goals (Miyake et al., 2000; Li et al., 2017). Important for postural control, executive function purportedly relies on the same frontal neural systems supporting motor control (Stuss, 2011).

As humans age, motor control increasingly relies on executive function (Duncan and Owen, 2000; Seidler et al., 2010; Al-Yahya et al., 2011, 2019; Holtzer et al., 2014), yet executive function also declines with age (Elderkin-Thompson et al., 2008; Grady, 2012; King et al., 2013; Yuan and Raz, 2014). Thus, in cognitive-motor dual-task situations involving executive function tasks, older adults’ restricted supply of executive function might result in greater performance costs compared to younger adults. In contrast, non-executive function tasks that do not rely as much on neural resources common to motor control may not result in comparable interference. This has not been directly tested in terms of balance performance; however, cognitive-motor dual-task interference from EF and non-EF tasks has been investigated in related motor domains such as gait (Beauchet et al., 2012). For example, one study measured gait for 20 younger adults and 17 older adults who completed single and dual-task walking scenarios. They found that EF-based secondary tasks slowed gait more non-EF tasks, and this EF-specific cognitive-motor dual-task interference was greater for older adults compared to younger adults (Walshe et al., 2015).

The current study builds on this prior research by investigating the specificity of cognitive-motor dual-task interference on balance using tablet-based executive function and non-executive function tasks, stable and unstable surfaces, and younger and older adults. Building on prior motor control research and leveraging a dominant model of EF (Miyake et al., 2000), we wanted to identify which combinations of cognitive load (i.e., non-EF demands, EF switching demands, EF updating demands, EF inhibition demands) and surface stability (i.e., stable, unstable) lead to the greatest impacts on sway, which could indicate situations where resources are most scarce (and thus, most shared). If secondary EF tasks lead to greater sway specifically on unstable surfaces compared to a non-EF task selectively, this would align more with models of limited attentional resources, such that performance declines as demand for a shared resource increases. This would also provide additional support for an overlap or taxation of concurrent processing between specific higher-level EFs and balance. On the other hand, if we see comparable impairment (i.e., greater sway) from the non-EF and EF tasks, then it’s possible that cognitive-motor dual-task impairment is not specific and perhaps instead results from general information-processing bottlenecks (Maylor and Wing, 1996; Dault et al., 2001a). Furthermore, we were interested in whether or not levels of cognitive-motor dual-task interference on balance would be comparable for young and older adults since prior research found greater impairments on EF tasks for older adults in a related motor domain (Walshe et al., 2015).

## Materials and Methods

### Participants

Based on prior research involving postural control, dual tasking, and young vs. older adults, our goal was to have at least 30 participants in each age group (Kerr et al., 1985; Yardley et al., 1999; Bergamin et al., 2014; Bohle et al., 2019; Hsiao et al., 2020). For the younger adults, we recruited 53 healthy adults (ages 18–35) and excluded 11 due to technical errors for a final sample of 42 younger adult participants (mean age = 23 years; 26F/16M). For the older adults, we recruited 37 healthy older adults (ages 60 or older (Walshe et al., 2015) and excluded 7 due to technical errors for a final sample of 30 older adult participants (mean age = 73 years; 27F/3M). For our convenience sample, we only recruited participants from the local community who could stand upright without assistance; had no balance impairments; were not taking any medication that could impact balance; were free from musculoskeletal and neurological disorders including dementia, depression, and other cognitive impairments; and had normal or corrected-to-normal vision. All participants provided written consent according to the Declaration of Helsinki, and our protocol was approved by the Tufts University IRB.

### Protocol

**FIGURE 1:**
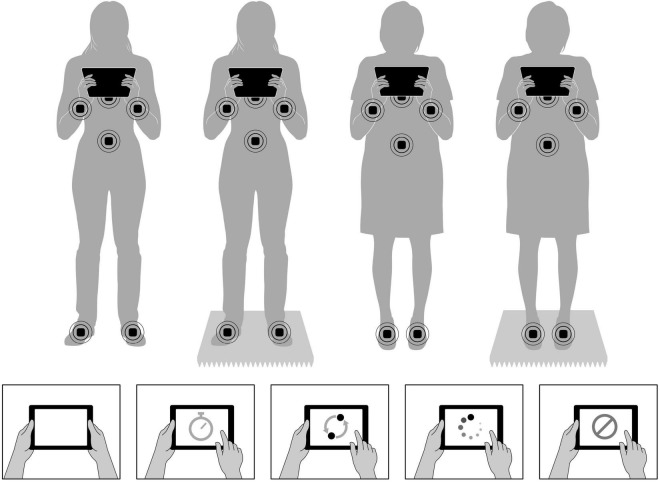
Top figures represent sensor placement for both age groups as they performed the various conditions on stable and unstable surfaces. Bottom images depict the five experimental conditions, which were Baseline (no task), non-EF processing speed, EF shifting, EF updating, and EF inhibition.

Participants completed a single session lasting approximately 1.5 h. After participants provided informed consent, we placed wearable sensors that measured postural sway on them. Next, participants completed baseline standing conditions on firm and foam surfaces while holding a tablet, and then they completed four tablet-based cognitive tasks on the firm surface and on the foam surface (Figure 1). We counterbalanced surface type blocks (i.e., firm surface first and foam surface second vs. foam surface first and firm surface second), and we randomized cognitive tasks within each surface type block. Finally, participants completed a brief survey at the end of the study before being debriefed and compensated for their time. The post experiment survey included two measures of interest. First, participants completed the Activities-Specific Balance Confidence Scale (ABC), which was a 16-item scale that assessed balance confidence when performing activities, such as walking up/down stairs or getting into/out of a car (Powell and Myers, 1995). We summed the scores and then divided by 16 for an overall balance confidence rating. Second, participants completed the short version of the Mobile Device Proficiency Questionnaire (MDPQ-16). The MDPQ-16 measures mobile device proficiency across eight domains, such as mobile device basics and data/file storage, with two items per domain (Roque and Boot, 2018). We averaged each of the subscales and then summed across the eight domains for a total score.

### Balance Procedure

We used six APDM Opal sensors to measure postural sway (Opal v2, APDM Inc., Portland, OR, United States). Participants wore these sensors on their feet, lumbar, sternum, and wrists (Deshmukh et al., 2012; Mancini et al., 2012; Martinez-Mendez et al., 2012; Doherty et al., 2017; Pissadaki et al., 2018; Morris et al., 2019). We measured center of pressure variability using the root mean square distance of sway acceleration (RMS Sway in m/s^2^), which quantified the magnitude of center of pressure displacements (Maki et al., 1994; Prieto et al., 1996; Rocchi et al., 2004; Mancini et al., 2011a; King et al., 2017), and we used APDM Mobility Lab v2.0 to process postural sway data (Mancini et al., 2011b). To ensure consistent foot placement across all trials, we used a foot placement template so that participants had approximately 10 cm between the right and left heel with a 30-degree outward foot rotation (Chiari et al., 2002; Morris et al., 2019). Participants stood on the firm floor of the lab for the stable conditions, and they stood on an Airex Elite foam balance pad (approximately 6 cm in height) for the unstable conditions (Šarabon et al., 2010; Gera et al., 2018).

### Computerized Cognitive Tasks

Participants completed tablet-based cognitive tasks administered through the mobile application BrainBaseline, which is a scientifically validated research tool (Lee et al., 2012; White et al., 2020; Ward et al., 2021). For our non-EF measure, participants completed a simple processing speed task in which they responded as quickly as possible whenever a circle appeared in the middle of the screen, and we used response time as our primary measure (Basner and Dinges, 2011). For our EF measures, participants completed a shifting task, an updating task, and an inhibition task (Miyake et al., 2000). For EF shifting, participants completed a task switching task in which they made parity (odd vs. even) or magnitude (less than 5 vs. greater than 5) judgments depending on the background color on each trial for a centrally presented number, and we calculated switch costs (i.e., the difference between correct switch trial RTs and correct repeat trial RTs) as our primary measure (Monsell, 2003). For EF updating, participants completed an N-back task which consisted of viewing a stream of sequentially presented numbers and determining whether the current number matched the number presented two trials previously, and we used 2-back accuracy as our primary measure (Owen et al., 2005). For EF inhibition, participants completed a Stroop task in which they responded to the font color of centrally presented words while ignoring the lexical content of the word, and we calculated the Stroop effect (i.e., the difference between correct incongruent RTs and correct congruent RTs) as our primary measure (Stroop, 1935; MacLeod, 1991). Full details on these four tasks have been described elsewhere (Lee et al., 2012).

## Results

### Balance Performance

For the postural sway data, we ran a 5 (Cognitive Load: Baseline vs. non-EF processing speed vs. EF shifting vs. EF updating vs. EF inhibition) × 2 (Surface Stability: firm vs. foam) × 2 (Age: younger vs. older) mixed model ANOVA (see Table 1 for postural sway descriptive statistics).

**TABLE 1:** Postural sway descriptive statistics.

		Age			
		Younger adults		Older adults	
Surface	Cognitive load	Mean	*SD*	Mean	*SD*
Firm	Baseline	0.026	0.013	0.022	0.010
	Processing speed	0.051	0.064	0.035	0.028
	EF Shifting	0.051	0.043	0.041	0.033
	EF Updating	0.051	0.091	0.037	0.033
	EF Inhibition	0.035	0.029	0.033	0.027
Foam	Baseline	0.042	0.015	0.065	0.041
	Processing speed	0.049	0.025	0.054	0.033
	EF Shifting	0.051	0.028	0.069	0.047
	EF Updating	0.051	0.023	0.057	0.042
	EF Inhibition	0.045	0.027	0.060	0.042

**FIGURE 2:**
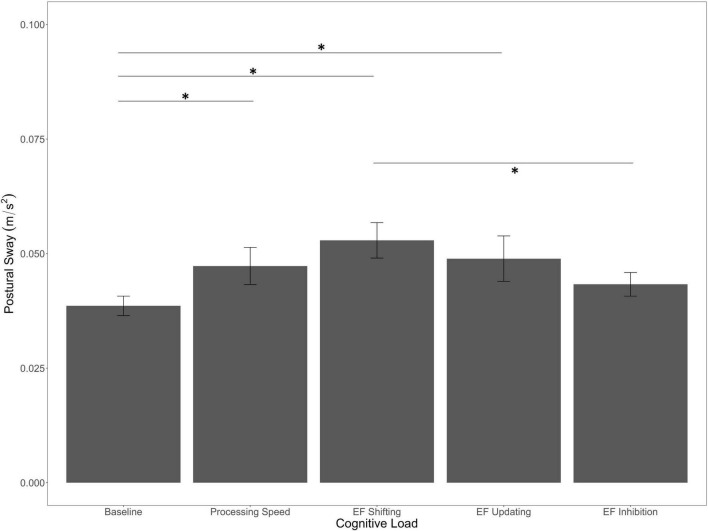
Main effect of cognitive load on postural sway. Error bars are ± SE. Significant differences are indicated by asterisks: *p < 0.05.

We found a main effect of Cognitive Load [*F*_(4, 280)_ = 3.89, *p* = 0.004, η^2^*_*p*_* = 0.05]. As seen in Figure 2, planned comparisons revealed that sway was significantly lower in the baseline condition compared to the EF shifting (*p* < 0.001) and the EF updating (*p* = 0.04) conditions, as well as the non-EF processing speed condition (*p* = 0.03). Although numerically in the expected direction, the difference between baseline and EF inhibition was not significant (*p* = 0.09). Interestingly, the non-EF processing speed condition did not differ from the EF conditions (*p’*s > 0.10), but the EF inhibition condition was significantly lower than the EF shifting condition (*p* < 0.05).

**FIGURE 3:**
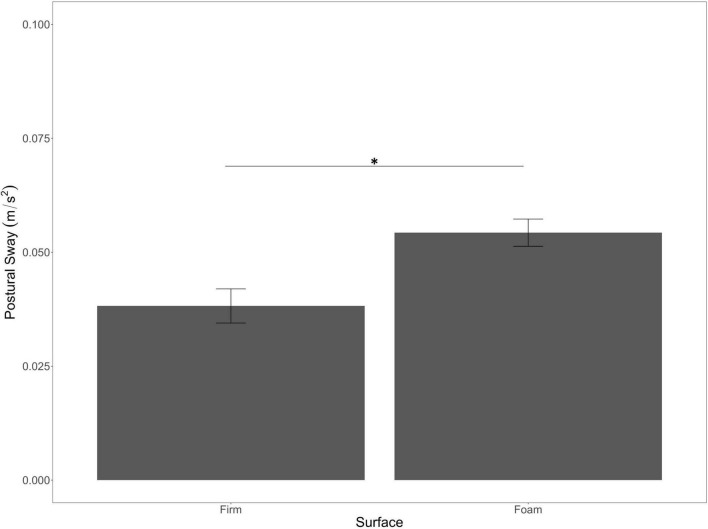
Main effect of surface stability on postural sway. Error bars are ± SE. Significant differences are indicated by asterisks: *p < 0.05.

We also found a main effect of Surface Stability [*F*_(1, 70)_ = 16.07, *p* < 0.001, η^2^*_*p*_* = 0.19] with significantly less sway in the firm condition compared to the foam condition (Figure 3).

**FIGURE 4:**
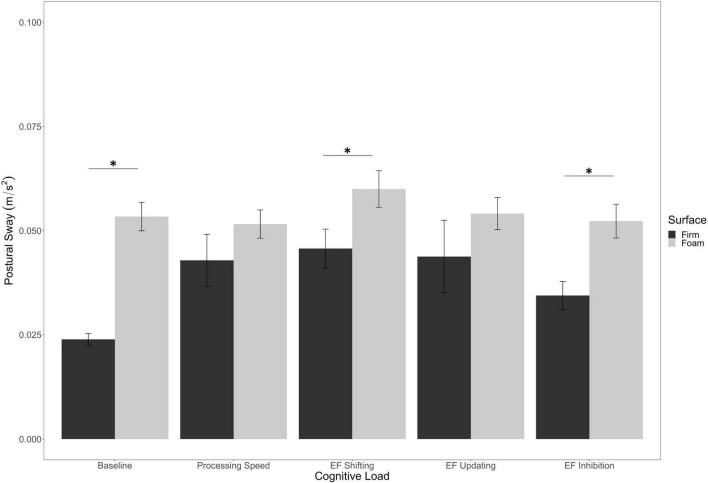
Interaction of surface stability by cognitive load on postural sway. Error bars are ± SE. Significant differences are indicated by asterisks: *p < 0.05.

In addition, we found an interaction between Cognitive Load and Surface Stability [*F*_(4, 280)_ = 2.84, *p* = 0.03, η^2^*_*p*_* = 0.04]. As seen in Figure 4, postural sway on the firm surface was significantly lower than on the foam surface for the Baseline (*p* < 0.001), EF shifting (*p* = 0.004), and EF inhibition (*p* = 0.001) conditions.

**FIGURE 5:**
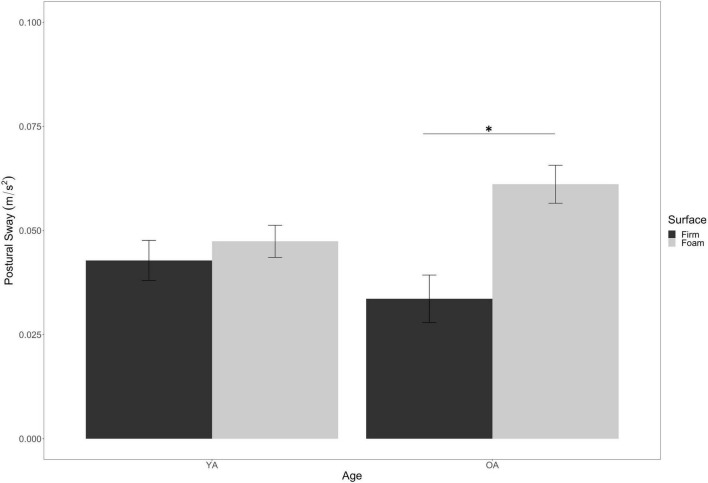
Interaction of surface stability by age on postural sway. Error bars are ± SE. Significant differences are indicated by asterisks: *p < 0.05.

Contrary to our expectations, we did not observe a main effect of Age [*F*_(1, 70)_ = 0.17, *p* = 0.69, η^2^*_*p*_* = 0.002] on sway, nor did we find an interaction between Age and Cognitive Load [*F*_(4, 280)_ = 1.53, *p* = 0.19, η^2^*_*p*_* = 0.02]. On the other hand, we observed an interaction between Age and Surface Stability [*F*_(1_, _70)_ = 8.09, *p* = 0.01, η^2^*_*p*_* = 0.10]. As seen in Figure 5, the older adult group had significantly higher sway on the foam surface compared to the firm surface (*p* < 0.001) whereas younger adults’ postural control did not differ across the two surface types (*p* = 0.37). Importantly, the older adult group did not differ from the younger adult group on the stable, firm surface (*p* = 0.22), whereas they did significantly differ from them on the unstable, foam surface (*p* = 0.03). We did not observe any other significant interactions among our factors (see Supplementary Materials for all statistical tests).

To recap our balance performance findings, we found main effects on sway from Cognitive Load and Surface Stability, as well as from an interaction between these factors. We also found an interaction between Age and Surface Stability in terms of sway, but no other analyses were significant.

### Cognitive Performance

For the cognitive data, we ran a series of mixed model ANOVAs for our different dependent measures with Surface Stability (firm vs. foam) as a within subjects factor and Age (younger vs. older) as a between subjects factor and used Bonferroni correction for an adjusted *p*-value of 0.0125 (see Table 2 for cognitive task descriptive statistics).

**TABLE 2:** Cognitive task descriptive statistics.

		Age			
		Younger adults		Older adults	
Surface	Cognitive load	Mean	*SD*	Mean	*SD*
Firm	Processing speed	357	47.3	427	103
	EF Shifting	186	185	302	338
	EF Updating	0.84	0.18	0.59	0.21
	EF Inhibition	146	82.4	348	203
Foam	Processing speed	360	53.6	441	108
	EF Shifting	179	234	296	260
	EF Updating	0.89	0.13	0.63	0.24
	EF Inhibition	135	93.3	353	197

**FIGURE 6:**
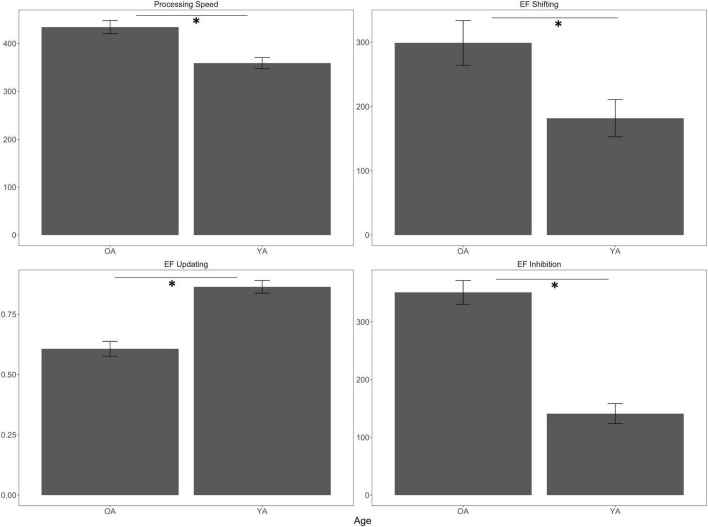
Main effects of age on cognitive task performance. Top left is Processing Speed (RT in ms). Top right is EF Shifting (switch costs in ms). Bottom right is EF Inhibition (Stroop effect in ms). Bottom left is EF Updating (2-back accuracy). Error bars are ± SE. Significant differences are indicated by asterisks: *p < 0.05.

For non-EF processing speed, we found a main effect of Age [*F*_(1, 70)_ = 17.40, *p* < 0.001, η^2^*_*p*_* = 0.20] with older adults producing significantly slower correct RTs compared to younger adults, which is seen in the top left of Figure 6. We did not find an effect of Surface Stability [*F*_(1, 70)_ = 3.29, *p* = 0.07, η^2^*_*p*_* = 0.05] nor an interaction between Age and Surface Stability in terms of non-EF processing speed [*F*_(1, 70)_ = 1.32, *p* = 0.25, η^2^*_*p*_* = 0.02].

For EF shifting, we found a main effect of Age [*F*_(1, 69)_ = 6.60, *p* = 0.01, η^2^*_*p*_* = 0.09] in which the older adults had higher switch costs compared to the younger adults, which is depicted in the top right of Figure 6. We did not find an effect of Surface Stability [*F*_(1, 69)_ = 0.03, *p* = 0.85, η^2^*_*p*_* = 0.001], nor did we find an interaction between Age and Surface Stability in terms of EF shifting [*F*_(1, 69)_ < 0.001, *p* = 0.99, η^2^*_*p*_* < 0.001].

For EF updating, we found a main effect of Age [*F*_(1, 70)_ = 40.50, *p* < 0.001, η^2^*_*p*_* = 0.37] with older adults exhibiting lower accuracy than younger adults, which is seen in the bottom left of Figure 6. We did not observe an effect of Surface Stability [*F*_(1, 70)_ = 4.42, *p* = 0.04, η^2^*_*p*_* = 0.06], nor did Age and Surface Stability interact in terms of EF updating [*F*_(1, 70)_ = 0.01, *p* = 0.92, η^2^*_*p*_* < 0.001].

Finally, for EF inhibition, we found an effect of Age [*F*_(1, 70)_ = 61.20, *p* < 0.001, η^2^*_*p*_* = 0.47] in which older adults had larger Stroop effects compared to younger adults, which is depicted in the bottom right of Figure 6. We did not find an effect of Surface Stability [*F*_(1, 70)_ = 0.01, *p* = 0.91, η^2^*_*p*_* < 0.001], nor did we find an interaction between Age and Surface Stability in terms of EF inhibition [*F*_(1, 70)_ = 0.13, *p* = 0.72, η^2^*_*p*_* = 0.002].

To recap our cognitive performance findings, we found effects of Age on our EF and non-EF tasks, but there were no effects of Surface Stability and no interactions between Age and Surface Stability on any of the cognitive measures (see Supplementary Materials for all statistical tests).

### Post-experiment Survey

Using the summary score on the ABC scale, we ran an independent samples *t*-test and found that younger adults reported significantly higher levels of confidence compared to the older adults [91% vs. 78%; *Welch’s t*(37) = 3.39, *p* = 0.002, Cohen’s *d* = 0.88]. Like with the ABC, we ran an independent samples *t*-test on the MDPQ total score and found that younger adults reported significantly higher levels of mobile device proficiency compared to the older adults [39 vs. 27; Welch’s *t*(27) = 5.62, *p* < 0.001, Cohen’s *d* = 1.52].

## Discussion

The current study is the first to examine the impact of cognitive demands of tablet-based EF and non-EF tasks among older and younger adults attempting to balance on surfaces of varying stability. We measured participants’ postural control using center of pressure displacements obtained from an array of wearable IMU sensors (Mancini et al., 2011b,^2012^; Deshmukh et al., 2012; Martinez-Mendez et al., 2012; Doherty et al., 2017), and we measured cognitive performance using response times and accuracy on the tablet tasks (Rossiter et al., 2017).

### Balance Performance

In general, more sway was observed on unstable surfaces compared to stable surfaces as evidenced by a main effect of surface stability, which aligns with prior research (Barbado Murillo et al., 2012; Tse et al., 2013). Furthermore, older adults showed this effect in a more exaggerated fashion compared to younger adults, which also replicates prior work and suggests that older adults’ motor control is more impacted by surface stability than younger adults (Abrahamová and Hlavacka, 2008; Boisgontier et al., 2013; Przysucha et al., 2020). One possible explanation for this comes from findings suggesting age-related declines in muscle mass needed for postural control, which can contribute to older adult risk of falling (Pijnappels et al., 2008). Another possible explanation for age-related sway differences in unstable conditions could stem from differences in balance confidence among older vs. younger adults. Indeed, the older adults in our study reported overall lower balance confidence than the younger adults. This suggests that they may have felt less equipped to perform the task, which may have introduced an additional cognitive demand for them, although this comes with some caveats. For instance, older adult balance performance was not actually correlated with balance confidence in our sample. That said, balance confidence scores were negatively correlated with postural sway in the EF updating and non-EF processing speed conditions for our younger adult group, although we refrain from making strong interpretations given that correlations tend not to reliably stabilize until much higher sample sizes (Schönbrodt and Perugini, 2013). Future studies should investigate possible role balance confidence may have on balance performance further with more comprehensive measures of balance confidence and with much larger samples.

In addition to being impacted by surface stability, postural control was impacted by cognitive load. Based on research in a motor ability related to postural control (i.e., gait; Walshe et al., 2015), we expected that EF and non-EF tasks would have differential effects on sway in part because of the purported overlap in neural resources for EF tasks and motor control, but this is not what we found. Instead, we observed that doing cognitive tasks in general on a tablet generated more sway compared to a baseline of holding an inactive tablet. Specifically, in our planned comparisons where we tested the difference in sway while performing each secondary task compared to sway during the baseline (single-task) condition, we observed significantly higher sway for all cognitive tasks relative to baseline except for EF inhibition, which instead trended in the same direction. This is surprising considering that our non-EF measure of processing speed supposedly imposes lower order cognitive demands than demands from EF measures (Maldonado et al., 2020); however, it’s also important to note that some prior models have suggested that balance measures are independent from gait measures (Horak et al., 2016), which could also account for differences between our findings on balance with EF/non-EF tasks and prior work on gait with EF/non-EF tasks.

Furthermore, we expected that given age-related declines in EF resources required for coordinating cognitive and postural demands, older adults might specifically struggle in cognitive-motor dual-task conditions involving EF demands compared to non-EF demands; however, the type of cognitive task demand did not interact with age. Instead, we only had a main effect of cognitive load. This suggests that introducing a cognitive demand via tablet tasks *generally* impacts postural control for both age groups; that is, all participants demonstrated poorer balance when actively engaged with a secondary task regardless of whether it was an EF task (Dault et al., 2001b; Bergamin et al., 2014). In addition, the overlap between neural resources needed for EF and motor control may not be the primary mechanism accounting for the present cognitive-motor dual-tasking effects, although more research with neuroimaging techniques (e.g., fNIRS) is needed to directly test such hypotheses related to limited cognitive resource overlap and multitasking bottlenecks (Rosso et al., 2017).

Despite the paucity of age effects, our results support the notion that postural control requires cognitive resources in general. In other words, engaging in a secondary cognitive task while standing upright generates cognitive-motor dual-task interference regardless of if the secondary cognitive task relies on EF or not and regardless of age group. This general interference account aligns more with one of two popular dual-tasking models. Bottleneck models posit that cognitive-motor dual-task interference results from serial processing restrictions when multiple tasks require similar information processing stages at the same time, which is not possible due to structural limitations (Pashler, 1994) or strategic control (Meyer and Kieras, 1997). Importantly, this model is somewhat agnostic in terms of the specific task combinations and would thus treat EF-related tasks similar to non-EF tasks. We found that both EF tasks and a non-EF task impacted balance compared to a single-task control condition, which is more compatible with the notion of general information processing bottlenecks.

Alternatively, according to capacity sharing models, cognitive-motor dual-task interference on balance results from limited-capacity parallel processing abilities to divide specific resources among the cognitive and motor tasks, which means that each task gets lower capacity leading to impairments (Navon and Gopher, 1979; Woollacott and Shumway-Cook, 2002). When similar resources are required for balance and EF-related cognitive task performance, interference should be greater than when less related attentional resources are required for balance and non-EF-related cognitive task performance. Once again, our results are less compatible with this theoretical account.

Regardless, more research is needed to further test assumptions of these models against a host of possible patterns observed in cognitive-motor dual-task research (Plummer et al., 2013; Bayot et al., 2018). For example, it is possible that when faced with competition for attentional resources, people must decide how to prioritize the two tasks, and in the current study, older and younger adults might have adopted similar task prioritization strategies, which is why we did not see more effects of age on balance (Yogev-Seligmann et al., 2012; Plummer and Eskes, 2015). Unfortunately, we did not think to ask participants if they prioritized balance performance over cognitive performance, nor do we know if these types of decisions are conscious and intentional. Future research manipulating participant instructions could help to more directly test the role that task prioritization might play in cognitive-motor dual-tasking settings.

### Cognitive Performance

Although our main focus was on balance performance, we also measured performance on computerized cognitive tasks. In terms of cognitive performance, we noted age effects on all cognitive tasks, where older adults had worse performance (e.g., lower accuracy, slower responses) compared to younger adults. This replicates prior results that suggest age-related declines in cognitive function (Elderkin-Thompson et al., 2008; Grady, 2012; Fraser and Bherer, 2013; King et al., 2013; Yuan and Raz, 2014). In our case all tasks were performed on a tablet platform, thus it is also possible that the age-related effects on the cognitive tasks are due, in part, to differences in mobile device proficiency across older and younger adults (Roque and Boot, 2018). That said, others have found age-related effects on cognitive tasks in cognitive-motor dual-task studies that did not use tablets (Prado et al., 2007), so more research is needed to better understand the impact of technology proficiency on balance.

## Conclusion

In today’s society, standing upright is rarely done without additional cognitive tasks and on completely stable, regular surfaces. Furthermore, normal aging often entails cognitive and physical changes that impact balance and the risk of falling, which is why we wanted to investigate balance for younger and older adults engaged in different types of cognitive tasks on stable and unstable surfaces. We chose to use three EF tasks compared to a non-EF task to better understand the specificity of cognitive-motor dual-task interference on balance, and we instead found general interference from the cognitive tasks that patterned similarly for both age groups. We used tablet-based cognitive tasks in part because of the increasing role of devices in daily life. Indeed, other cognitive-motor dual-tasking studies have found that using mobile devices can impact postural control for both younger and older adults (Cho et al., 2014; Nurwulan et al., 2015; Laatar et al., 2017; Bruyneel and Duclos, 2020; Hsiao et al., 2020; Onofrei et al., 2020). Future work with larger samples is needed to extend investigations into the specificity of cognitive-motor dual-task interference on balance to more realistic tasks people complete on mobile devices.

## Data Availability Statement

The raw data supporting the conclusions of this article will be made available by the authors, without undue reservation.

## Ethics Statement

The studies involving human participants were reviewed and approved by the Tufts University IRB. The patients/participants provided their written informed consent to participate in this study.

## Author Contributions

NW and EH: conceptualization. AM, VU, and CR: data curation and investigation. NW and TW: formal analysis. NW, EH, VU, CR, and EM: methodology. NW and EM: resources and supervision. NW: writing—original draft. NW, EH, AM, VU, CR, TW, and EM: writing—review and editing. All authors have read and agree to the published version of the manuscript.

## Conflict of Interest

The authors declare that the research was conducted in the absence of any commercial or financial relationships that could be construed as a potential conflict of interest.

## Publisher’s Note

All claims expressed in this article are solely those of the authors and do not necessarily represent those of their affiliated organizations, or those of the publisher, the editors and the reviewers. Any product that may be evaluated in this article, or claim that may be made by its manufacturer, is not guaranteed or endorsed by the publisher.

## Supplementary Material

Supplementary Data Sheet 1 De-identified data.
