# Supplementary material for: The Specificity of Cognitive-Motor Dual-Task Interference on Balance in Young and Older Adults
Source: Front Aging Neurosci. 2022 Jan 11;13:804936. doi: 10.3389/fnagi.2021.804936 (PMC8786904; doi:10.3389/fnagi.2021.804936)
Supplement: Supplementary file 2 [file Table_1.DOCX]

Supplemental Table 1: Inferential statistics for balance performance

|  | ***F*** | ***df*** | ***p*** | ***η²_p_*** |
| --- | --- | --- | --- | --- |
| Surface Stability | 16.07 | 1,70 | < .001 | 0.19 |
| Cognitive Load | 3.89 | 4,280 | 0.00 | 0.05 |
| Surface Stability x Cognitive Load | 2.84 | 4,280 | 0.03 | 0.04 |
| Age | 0.17 | 1,70 | 0.69 | 0.00 |
| Age x Surface Stability | 8.09 | 1,70 | 0.01 | 0.10 |
| Age x Cognitive Load | 1.53 | 4,280 | 0.19 | 0.02 |
| Age x Surface Stability x Cognitive Load | 0.27 | 4,280 | 0.90 | 0.00 |

Supplemental Table 2: Inferential statistics for cognitive performance

|  |  | ***F*** | ***df*** | ***p*** | ***η²_p_*** |
| --- | --- | --- | --- | --- | --- |
| Non-EF Processing Speed | Surface Stability | 3.29 | 1,70 | 0.07 | 0.05 |
|  | Age | 17.40 | 1,70 | < .001 | 0.20 |
|  | Age x Surface Stability | 1.32 | 1,70 | 0.25 | 0.02 |
| EF Shifting | Surface Stability | 0.03 | 1,69 | 0.85 | < .001 |
|  | Age | 6.60 | 1,69 | 0.01 | 0.09 |
|  | Age x Surface Stability | < .001 | 1,69 | 0.99 | < .001 |
| EF Updating | Surface Stability | 4.42 | 1,70 | 0.04 | 0.06 |
|  | Age | 40.50 | 1,70 | < .001 | 0.37 |
|  | Age x Surface Stability | 0.01 | 1,70 | 0.92 | < .001 |
| EF Inhibition | Surface Stability | 0.01 | 1,70 | 0.91 | < .001 |
|  | Age | 61.20 | 1,70 | < .001 | 0.47 |
|  | Age x Surface Stability | 0.13 | 1,70 | 0.72 | 0.00 |
